# Phosphatidylinositol glycan anchor biosynthesis, class C is a prognostic biomarker and correlates with immune infiltrates in hepatocellular carcinoma

**DOI:** 10.3389/fgene.2022.899407

**Published:** 2022-08-19

**Authors:** Qian Zhao, Chuan Shen, Junwei Wei, Caiyan Zhao

**Affiliations:** ^1^ Office of Quality Management and Control in Healthcare, The Third Hospital of Hebei Medical University, Shijiazhuang, China; ^2^ Department of Infectious Disease, The Third Hospital of Hebei Medical University, Shijiazhuang, China; ^3^ Department of Gastroenterology, The First Hospital of Handan City, Handan, China

**Keywords:** PIGC, hepatocellular carcinoma, prognosis, pathways, tumor-infiltrating

## Abstract

**Background and aims:** The exact function of Phosphatidylinositol Glycan Anchor Biosynthesis, Class C *(PIGC)* gene has yet to be elucidated. In the study, we attempted to clarify the correlations of *PIGC* to prognosis and tumor-infiltrating lymphocytes in hepatocellular carcinoma (HCC).

**Methods:** *PIGC* expression was analyzed *via* the Oncomine database, Gene Expression Profiling Interactive Analysis, Hepatocellular carcinoma data base, Human Protein Atlas database and Tumor Immune Estimation Resource (TIMER). We showed the correlation of *PIGC* with the clinical characteristics using UALCAN. We evaluated the influence of *PIGC* on clinical prognosis using Kaplan-Meier plotter databases. And co-expressed genes with *PIGC* and its regulators were identified using LinkedOmics. The correlations between *PIGC* and cancer immune infiltrates were investigated *via* TIMER. We analyzed the drug sensitivity and immunotherapy response via R package.

**Results:** *PIGC* was found up-regulated in tumor tissues in multiple HCC cohorts, also increased in HCC patient with different clinical characteristics. High *PIGC* expression was associated with poorer overall survival. *PIGC* expression showed a strong positive association with the expression of ACBD6, a strong negative association with AGXT212. The cell components and distribution in treatment and non-treatment of HCC patients were quite distinct, which may reveal the relationship between the immunotherapy with tumor microenvironment. Notably, *PIGC* expression was positively correlated with infiltrating levels of immune cells.

**Conclusion:** These findings suggest that *PIGC* is correlated with prognosis and immune infiltrating in HCC, which can be used as a prognostic biomarker for determining prognosis, laying a foundation for further study of the immune regulatory role of *PIGC* in HCC.

## Introduction

According to the International Agency for Research on Cancer (IARC), hepatocellular carcinoma (HCC) accounts for approximately 90% of all cases of primary liver cancer and is the third leading cause of cancer related deaths (8.3% of the total number of deaths by cancer) (Cancer IAfRo, 2020). Due to the high rate of recurrence and metastasis, the 5-year survival rate for patients with advanced HCC is poor. Existing drugs are all associated with unsatisfactory efficacy; this is due to a combination of factors spanning an array of different clinical and biological behaviors and the development of anti-HCC drug resistance. There is a clear need to identify novel targets that are critical for carcinogenesis and exploit these as therapeutic interventions to sustain the numerous innovations we have witnessed in patient care. Although previous studies have provided important insights relating to the molecular mechanisms of HCC, our understanding of HCC is still lacking. Several pathways and processes have been implicated in the progression of HCC, including telomere maintenance, the Wnt/β-catenin pathway, the inactivation of p53 and alterations in the cell cycle, chromatin remodeling complexes and epigenetic regulators (Llovet et al., 2016). The molecular mechanisms underlying tumor formation and progression are poorly understood, thus further complicating the effective treatment of HCC. It is also imperative to investigate the molecular mechanisms underlying HCC to develop new methods for the prevention and therapy of HCC.

Glycosylphosphatidylinositol (GPI) anchoring is a post-translational modification that tethers proteins to the plasma membrane and is thought to play a role in protein sorting and trafficking. In mammals, there are over 150 GPI-anchor proteins (GPI-APs), including receptors, adhesion molecules and enzymes. GPI-APs are functionally diverse and play vital roles in signal transduction, immune response, cancer cell invasion and metastasis, as well as the pathophysiology of parasites (McKean and Niswander, 2012; Tsai et al., 2012; Park et al., 2013). GPI-AP synthesis is divided into three steps: GPI biosynthesis, protein-attachment to GPI (trans-amidation), and GPI-AP remodeling. This complex process is performed by 15 enzymes that are encoded by at least 26 phosphatidylinositolglycan (*PIG*) genes, of which 22 *PIG* genes are involved in the GPI-core preassembly and following attachment to the protein (Kinoshita, 2014; Guerrero et al., 2019). In the current study, we focus on the Phosphatidylinositol Glycan Anchor Biosynthesis, Class C *(PIGC)* gene which encodes for a subunit of GPI-GlcNAc transferase (GPI-GnT) enzyme. The *PIGC* gene is located on chromosome 1 (1q24.3); the translated protein is composed of 297 amino acids and is localized to the endoplasmic reticulum (ER). Even though the exact function of *PIGC* has yet to be elucidated, it appears to be a crucial subunit of GPI-GnT, since *PIGC* effectively rescues yeast homologue GPI2 absence (GPI biosynthesis defect class C). *PIGC* is over-expressed in breast cancer according to the Human Protein Atlas and cancer genomics databases (Ponten et al., 2009; Gao et al., 2013; Armanios et al., 2018). Finally, previous research has identified single nucleotide polymorphisms (SNPs) in the *PIGC* gene in patients with HCC, which screened the genes with differentially expressed splicing variants between HCC and adjacent non-cancerous tissues, *PIGC* included (Liu et al., 2017).

In this present study, we comprehensively analyzed the expression of *PIGC* and correlated this data with the prognosis of cancer patients in databases such as Oncomine, Gene Expression Profiling Interactive Analysis (GEPIA), UALCAN and Kaplan-Meier plotter (KM plotter). We also investigated the correlation between *PIGC* and tumor-infiltrating immune cells in different tumor microenvironments via the Tumor Immune Estimation Resource (TIMER). The findings in this report shed light on the important role of *PIGC* in colorectal cancer with HCC and highlights the potential relationship and an underlying mechanism between *PIGC* and tumor-immune interactions.

## Materials and methods

### The expression of *PIG* genes in various cancers

GEPIA (http://gepia.cancer-pku.cn/) is a newly developed interactive web server for analyzing the RNA sequencing expression data of 9,736 tumors and 8,587 normal samples from the TCGA and the GTEx projects. In this study, we analyzed the expression levels of *PIG* genes in various cancers using the GEPIA.

### The expression of *PIGC* in HCC and normal tissues

Hepatocellular carcinoma database (HCCDB) (http://lifeome. net/database/hccdb) serves as a one-stop online resource for exploring HCC gene expressions. And Oncomine contains 65 gene expression datasets comprising nearly 48 million gene expression measurements form over 4,700 microarray experiments. The expression levels of *PIGC* mRNA in HCC and normal tissues were analyzed in the HCCDB and Oncomine database. The copy number of *PIGC* in HCC and normal tissues was also analyzed in the Oncomine database.

The Human Protein Atlas (HPA) (https://www.proteinatlas.org/) is a program with the aim to map all the human proteins in cells, tissues, and organs using an integration of various omics technologies, including antibody-based imaging, mass spectrometry-based proteomics, transcriptomics, and systems biology. The expression levels of *PIGC* protein in HCC and normal tissues were analyzed in immunohistochemistry images retrieved from the HPA.

### The relationship between *PIGC* expression and the clinical characteristics of patients with HCC

UALCAN is a user-friendly, interactive web resource for analyzing cancer transcriptome data. In this study, we performed sub-group analysis of the multiple clinico-pathological features of The Cancer Genome Atlas Liver Hepatocellular Carcinoma (TCGA-LIHC) samples using the UALCAN database (http://ualcan.path.uab.edu/index.html). *PIGC* mRNA expression in cancer was separately analyzed with patient characteristics of sample types, individual cancer stage, age, histological subtype, race, gender, and tumor grade compared to the normal liver tissue expression. The statistical analysis between two variables was performed by unpaired *t*-test, and one-way ANOVA analysis was performed for more than two variables.

### The relationship between *PIGC* expression and the survival of patients with HCC

The KM plotter can investigate the effect of 30,000 genes on survival in 21 different types of cancer, including breast, lung, ovarian, gastric, liver and pan-cancer. In the present study, the correlation between *PIGC* expression and survival in HCC patients was analyzed by the KM plotter (http://kmplot. com/analysis/). We also computed the hazard ratio (HR) with 95% confidence intervals and log-rank *p*-values.

### The profile of *PIGC* mutations in HCC

The cBio Cancer Genomics Portal (the cBio portal) (http://cbioportal.org) is an open-access resource for interactive exploration of multidimensional cancer genomics data sets, currently providing access to data from more than 5,000 tumor samples from 20 cancer studies. The profile of *PIGC* mutations in HCC was analyzed using the cBio Portal tool.

### Performed a co-expression networks in HCC

LinkedOmics is a publicly available portal that includes multi-omics data from all 32 the Cancer Genome Atlas(TCGA) cancer types. This portal also includes mass spectrometry-based proteomics data generated by the Clinical Proteomics Tumor Analysis Consortium (CPTAC) for TCGA breast, colorectal and ovarian tumors. In the present study, we used the online LinkedOmics database to identify genes that were significantly correlated with *PIGC* in HCC. Spearman’s method was used to determine the correlation. Further, we selected enrichment analysis to reveal the co-altered genes signaling pathway in HCC of the LinkedOmics. Program is running, and 1,000 simulations were performed. We used overrepresentation enrichment analysis (ORA) and gene set enrichment analysis (GSEA) as tools, and selected criteria FDR<0.05, positively correlated.

### Correlation between *PIGC* and the tumor immune microenvironment

Tumor Immune Single Cell Hub (TISCH) (http://tisch.comp-genomics.org) collected data from Gene Expression Omnibus (GEO) and ArrayExpress to formulate its scRNA-seq atlas. TISCH includes 79 databases and 2,045,746 cells from both tumor patients and healthy donors. The dataset was uniformly processed to enable clarifying components of the TME at both single-cell and annotated cluster levels. In this work, we derived from TISCH to decipher the Tumor Immune Microenvironment (TME) heterogeneity of HCC sites at single cell level, and found the correlation between *PIGC* and the TME.

### The correlation of *PIGC* expression with immune infiltration level in HCC

TIMER is a comprehensive resource for the systematic analysis of immune infiltrates across a diverse array of cancer types (https://cistrome.shinyapps.io/timer/). The TIMER database includes 10,897 samples across 32 cancer types from TCGA to estimate the abundance of immune infiltrates. We analyzed the expression levels of *PIGC* in different types of cancer and the correlation of *PIGC* expression with the abundance of immune infiltrates, including B cells, CD4^+^ T cells, CD8^+^ T cells, neutrophils, macrophages, and dendritic cells, via gene modules. In addition, correlations between *PIGC* expression and gene markers of tumor-infiltrating immune cells were investigated via correlation modules. The gene markers of tumor-infiltrating immune cells included markers of CD8+T cells, T cells (general), B cells, monocytes, tumor-associated macrophages (TAMs), classically activated macrophages (M1 macrophages), alternatively activated macrophages (M2 macrophages), neutrophils, natural killer (NK) cells, dendritic cells (DCs), T-helper 1 (Th1) cells, T-helper 2 (Th2) cells, follicular helper T (Tfh) cells, T-helper 17 (Th17) cells, Tregs, and exhausted T cells. These gene markers were described in previous studies (Sousa and Maatta, 2016; Danaher et al., 2017; Siemers et al., 2017).

The online database GEPIA was used to further confirm the significantly correlated genes in TIMER. Gene expression correlation analysis was performed for given sets of TCGA expression data. Analysis involved both tumor and normal tissue datasets.

### Drug sensitivity and immunotherapy response analysis

CellMiner is a web resource that provides tools for the acquisition and analysis of quality-controlled NCI-60 data. CellMiner tools allowed rapid data retrieval of transcripts for 22,379 genes and 360 microRNAs along with activity reports for 20,503 chemical compounds including 102 drugs approved by the US Food and Drug Administration. We got the RNA-sequencing and compound activity, and analyzed the prediction of drug sensitivity via R package.

Also, RNA-sequencing expression profiles and corresponding clinical information for HCC were downloaded from the TCGA dataset to analyze the immunotherapy response. Potential immune checkpoint blocksde (ICB) response was predicted with TIDE algorithm.

### Statistical analysis

Gene expression data from the Oncomine database were analyzed using *p*-values, fold changes, and ranks. Survival curves were produced by KM plots and the GEPIA database. Correlations of gene expression or immune signature score were determined in the TIMER and GEPIA databases using Spearman’s correlation analysis. The threshold of *p* < 0.05 indicates the significance of correlation.

## Results

### The expression of *PIG* genes in various cancers

**FIGURE 1:**
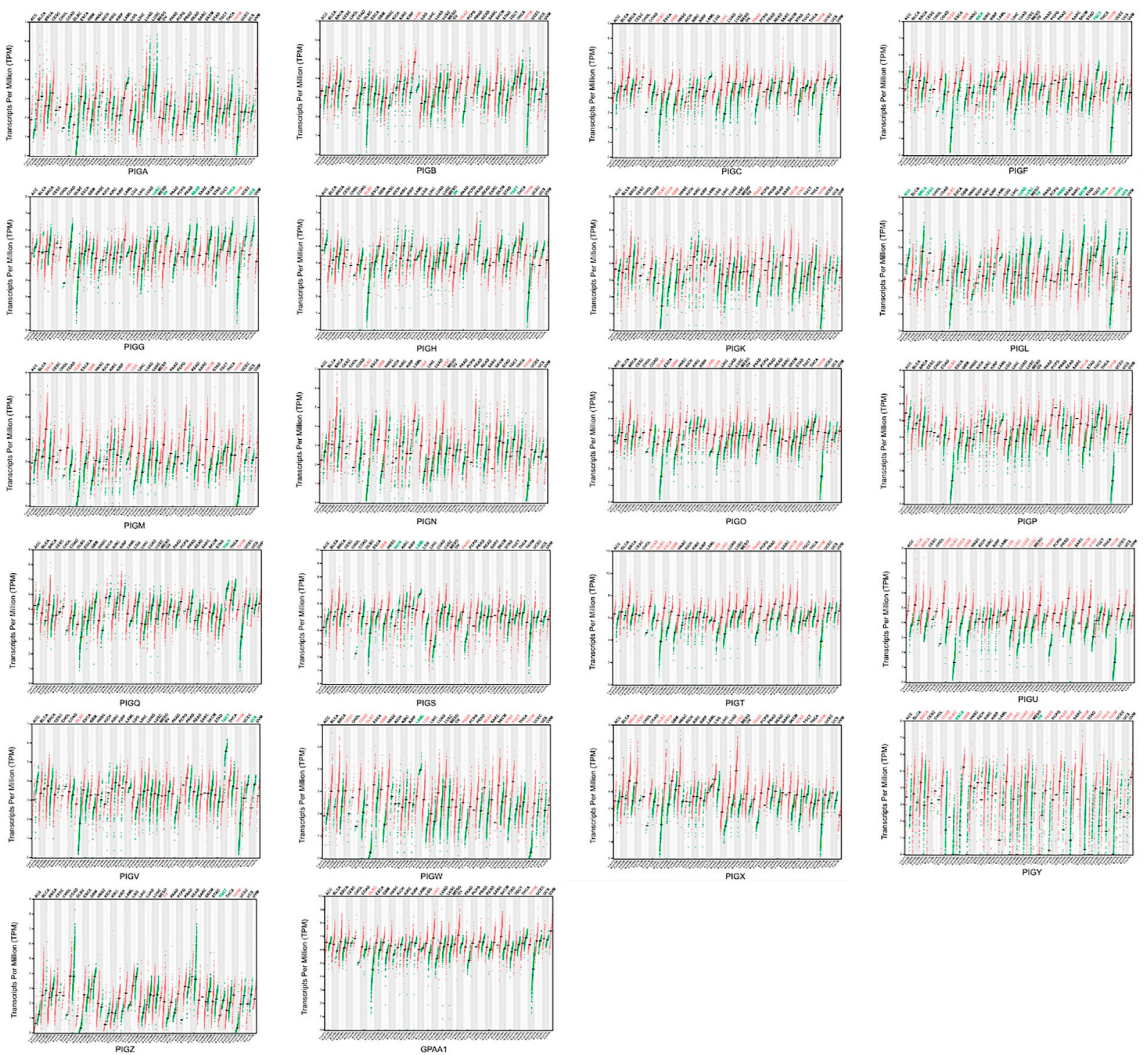
*PIG* genes expression in various cancers.

We analyzed the expression levels of *PIG* genes in 33 different types of human cancer and their paired normal tissues and compared this data with expression data retrieved from a combination of TCGA and GTEx data using GEPIA tools. Of the 22 genes, *PIGC*、PIGT、PIGU、PIGY、GPAA1 exhibited significantly higher expression level of 33 cancer types(Figure 1).

### The expression of *PIGC* mRNA and protein in HCC

**FIGURE 2:**
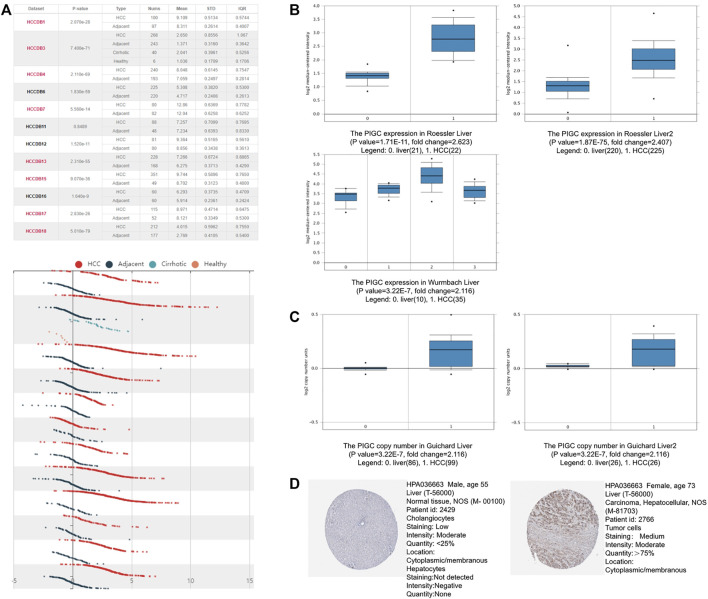
The *PIGC* Expression in HCC **(A)** The mRNA expression levels of *PIGC* in HCC using the HCCDB database. **(B)** the mRNA expression of *PIGC* in Roessler Liver dataset, Roessler Liver two dataset and Wurmbach Liver dataset. **(C)** the copy number of *PIGC* in Guichard Liver dataset and Guichard Liver two dataset. **(D)** the *PIGC* protein expression of tumor samples in the immunohistochemistry data from The Human Protein Atlas.

HCCDB database analysis demonstrated the increased expression levels of *PIGC* mRNA in 11 cohorts of 12 HCC patient cohorts when compared to narmal tissues (Figure 2A). Then, we examined the mRNA expression of *PIGC* in different datasets. An Oncomine box plot showed that the *PIGC* mRNA levels were significantly higher than those in normal tissues by the data from Roessler Liver (*p* < 0.001), Roessler Liver2 (*p* < 0.001), Wurmbach Liver (*p* < 0.001) (Figure 2B), and copy number in HCC were also significantly higher than those in normal tissues by the data from Guichard Liver (*p* < 0.001), Guichard Liver2 (*p* < 0.001) (Figure 2C). Next, we sought to verify this trend at the protein level between HCC and normal tissue. Analysis of immunohistochemistry data from The Human Protein Atlas showed that 10 out of 11 HCC patient samples exhibited moderate or weak staining signals, whereas normal Hepatocytes in healthy liver tissue did not exhibit detectable levels of *PIGC* expression (Figure 2D).

### The association between *PIGC* expression and the clinical characteristics of patients with HCC

**FIGURE 3:**
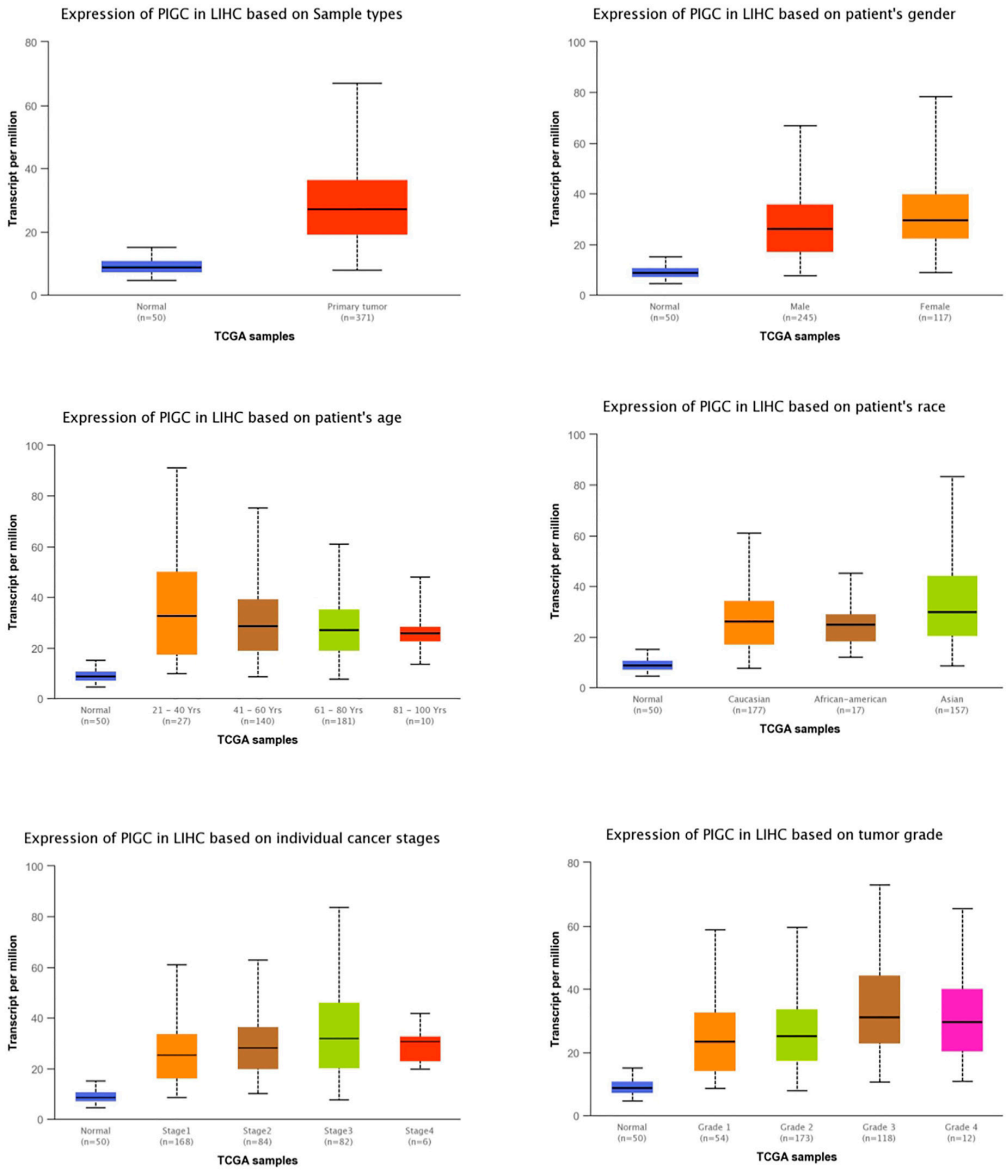
The relationship of *PIGC* Expression with Clinical Characteristics of HCC Patients based on gender, age, and other criteria (UALCAN).

Next, we investigated the association between *PIGC* mRNA expression and the clinical characteristics of HCC patients using TCGA data and UALCAN. Compared to the normal tissue, the expression levels of *PIGC* were higher regardless of the type of sample (normal, primary tumor), patient gender (male, female), patient age (20–40 years, 41–60 years, 61–80 years, and 81–100 years), patient race (Caucasian, African-American, and Asian), individual cancer stages (S1, S2, S3, and S4), and tumor grade (G1, G2, G3, G4). We found that *PIGC* expression was highest in the early age group (21–40 years) of HCC patients when compared to any other age group and that *PIGC* expression in the females and the Asian was higher than all other genders and races. The expression levels of *PIGC* mRNA were also higher in all individual cancer stages, but particularly in stage 3. In terms of tumor grading, the expression of *PIGC* was significantly up-regulated in all tumor grades, particularly grade 3 (Figure 3).

### The correlation between *PIGC* expression and the survival of patients with HCC

**FIGURE 4:**
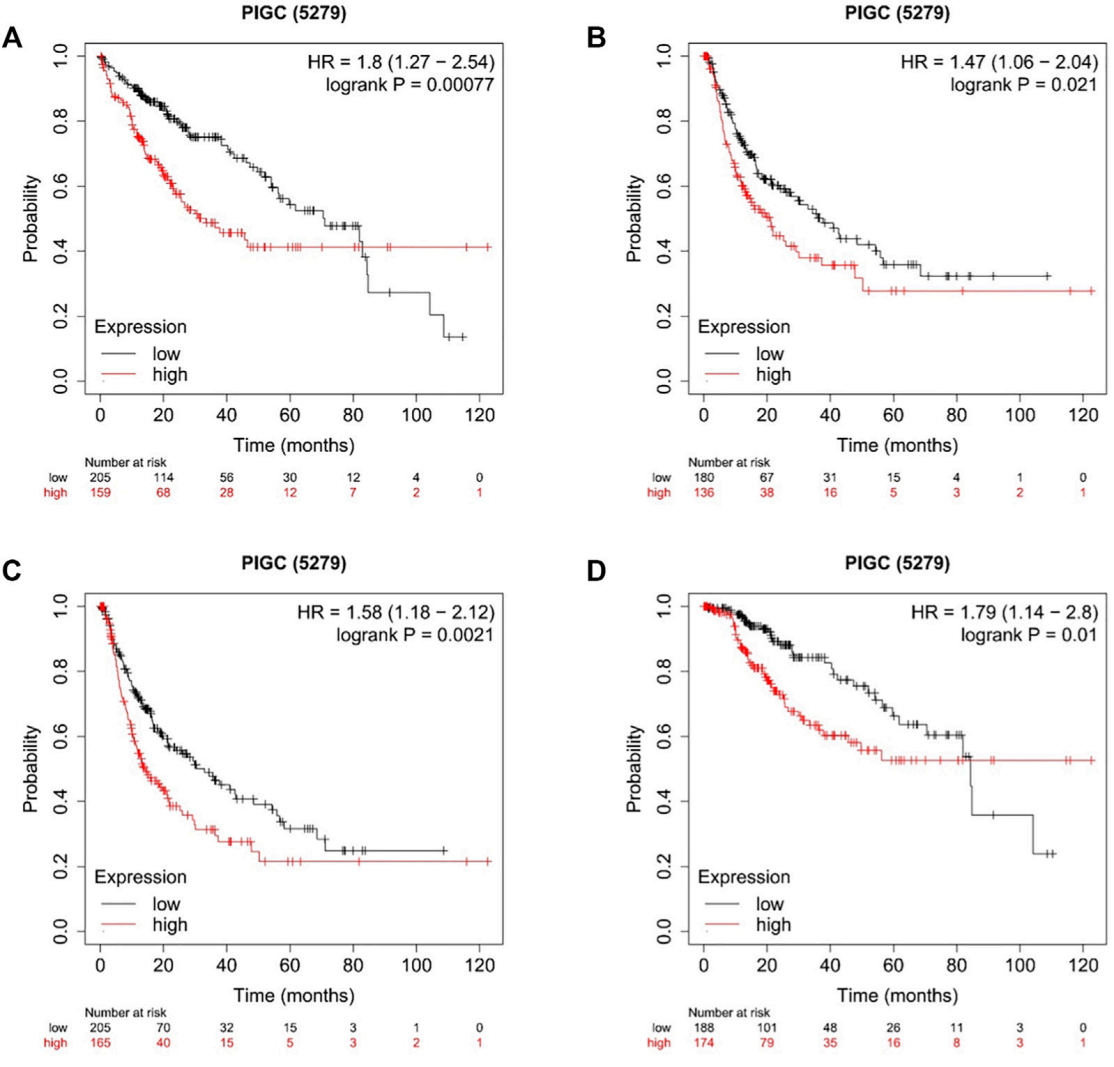
Kaplan–Meier plot of the Correlation of *PIGC* Expression and Patient Survival in HCC **(A)** Overall survival (OS). **(B)** Recurrence free survival (RFS). **(C)** Progression Free Survival (PFS). **(D)** Disease Free Survival (DFS).

To better understand the relevance and underlying mechanisms of *PIGC* expression in cancer, we investigated the specific relationship between *PIGC* expression and the clinical characteristics of HCC patients in KM plotter databases. The patients were separated into two groups according to the expression levels of *PIGC* mRNA. Generally, Kaplan-Meier analysis showed that the high *PIGC* expression group had a significantly shorter overall survival (OS) (log rank test, *p* = 0.00077) (Figure 4A), recurrence free survival (RFS) (log rank test, *p* = 0.021) (Figure 4B), progression free survival (PFS) (log rank test, *p* = 0.0021) (Figure 4C), and disease free survival (DFS) (log rank test, *p* = 0.01) (Figure 4D). The overexpression of *PIGC* was significantly associated with a worse OS and PFS in male and female patients (*p* < 0.05). Furthermore, high levels of *PIGC* mRNA expression were correlated with a worse OS in stage 1 and stage 3, but were not associated with the PFS of stage 1, stage 2, and stage 3. Specifically, high levels of *PIGC* mRNA expression were correlated with a worse OS in grade 1, grade 2 and grade 3, with a worse PFS for grade 1and grade 2, but were not associated with the PFS of grade 3 (Table 1).

**TABLE 1:** Correlation of PIGC mRNA expression and prognosis in hepatocellular carcinoma with different clinicopathological factors by Kaplan-Meier plotter.

Clinicopathological factors	Overall survival			Progression-free survival		
	N	Hazard ration	*p*-value	N	Hazard ration	*p*-value
Sex						
Female	118	2.46 (1.31–4.62)	0.0039	120	1.84 (1.08–3.13)	0.022
Male	246	2.12 (1.35–3.34)	0.00085	246	1.78 (1.24–2.55)	0.0014
Stage						
1	170	2 (1.08–3.69)	0.024	170	1.54 (0.94–2.54)	0.087
2	83	1.29 (0.59–2.83)	0.53	84	1.59 (0.85–3)	0.14
1 + 2	253	1.59 (0.98–2.56)	0.056	254	1.59 (1.08–2.32)	0.016
3	83	2.58 (1.39–4.76)	0.0018	83	1.72 (0.97–3.04)	0.061
4	4	-	-	5	-	-
3 + 4	87	2.68 (1.47–4.89)	0.00088	88	1.77 (1.02–3.07)	0.039
Grade						
1	55	2.99 (1.16–7.7)	0.018	55	4.81 (1.71–13.49)	0.0013
2	174	2.02 (1.2–3.39)	0.0068	175	2.18 (1.39–3.42)	0.00054
3	118	2.37 (1.1–5.12)	0.024	119	1.47 (0.84–2.57)	0.17
4	12	-	-	12	-	-
AJCC_T						
1	180	2.04 (1.13–3.66)	0.015	180	1.61 (0.99–2.61)	0.053
2	90	1.3 (0.63–2.7)	0.48	92	1.42 (0.8–2.54)	0.23
3	78	2.59 (1.39–4.86)	0.0021	78	1.92 (1.04–3.54)	0.033
4	13	-	-	13	-	-
Vascular invasion						
Micro	90	1.85 (0.85–4.03)	0.12	91	1.88 (1.05–3.36)	0.03
Macro	16	-	-	16	-	-
None	203	2.34 (1.39–3.94)	0.0011	204	1.85 (1.17–2.93)	0.0076
Race						
White	181	1.73 (1.09–2.74)	0.019	183	1.8 (1.21–2.68)	0.0031
Black or african american	17	-	-	17	-	-
Asian	155	3.17 (1.52–6.6)	0.0011	155	1.66 (1.03–2.7)	0.037
Alcohol consumption						
Yes	115	3.18 (1.65–6.13)	3.00E-04	115	2.04 (1.21–3.43)	0.0061
None	202	1.6 (1.01–2.53)	0.045	204	1.49 (0.98–2.26)	0.059
Hepatitis virus						
Yes	150	1.53 (0.8–2.94)	0.2	152	0.68 (0.42–1.09)	0.11
None	167	3.09 (1.92–4.98)	1.40E-06	167	2.43 (1.55–3.82)	7.70E-05

Bold values indicate *p* < 0.05.

### *PIGC* mutation in HCC

**FIGURE 5:**
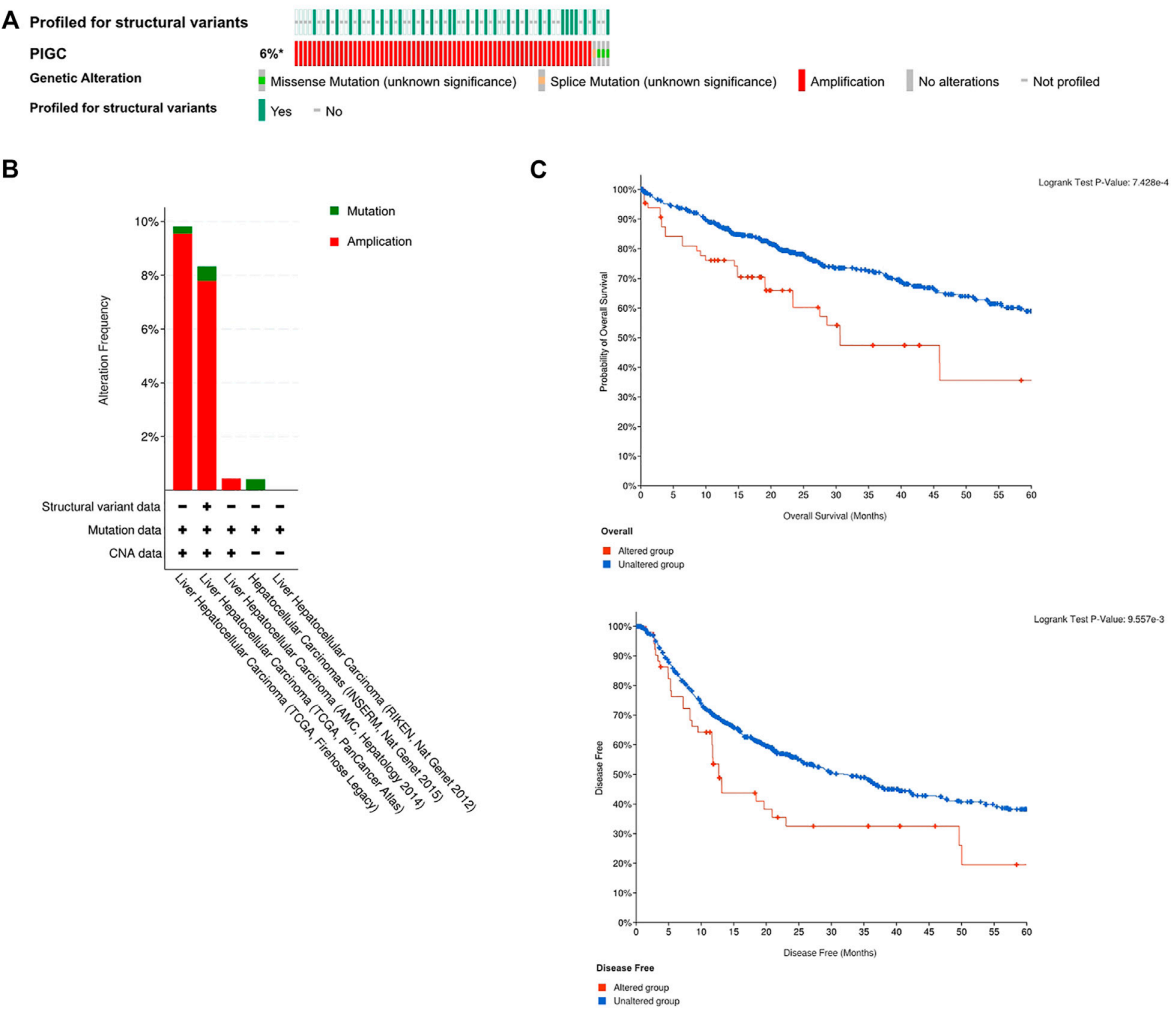
The profile of the *PIGC* mutation **(A)** The profile of the *PIGC* mutation of the Cbio Portal. **(B)** The alteration frequency of different HCC datasets. **(C)** The Kaplan–Meier plot of the Correlation of *PIGC* Expression and mutation.

We logged on to the cBio Portal website to explore the mutation rate of *PIGC*. As shown in Figure 5A, the mutation rate of *PIGC* is 6% in patients with HCC based on the data from TCGA-LIHC. Moreover, the styles of genetic alteration included the missense mutation, splice mutation and amplification(Figures 5A, B). Furthmore, Kaplan-Meier analysis showed that the *PIGC* mutation group had a significantly shorter OS (log rank test, *p* < 0.001) and DFS (log rank test, *p* < 0.01) (Figure 5C).

### The co-expression of genes with *PIGC* in HCC

**FIGURE 6:**
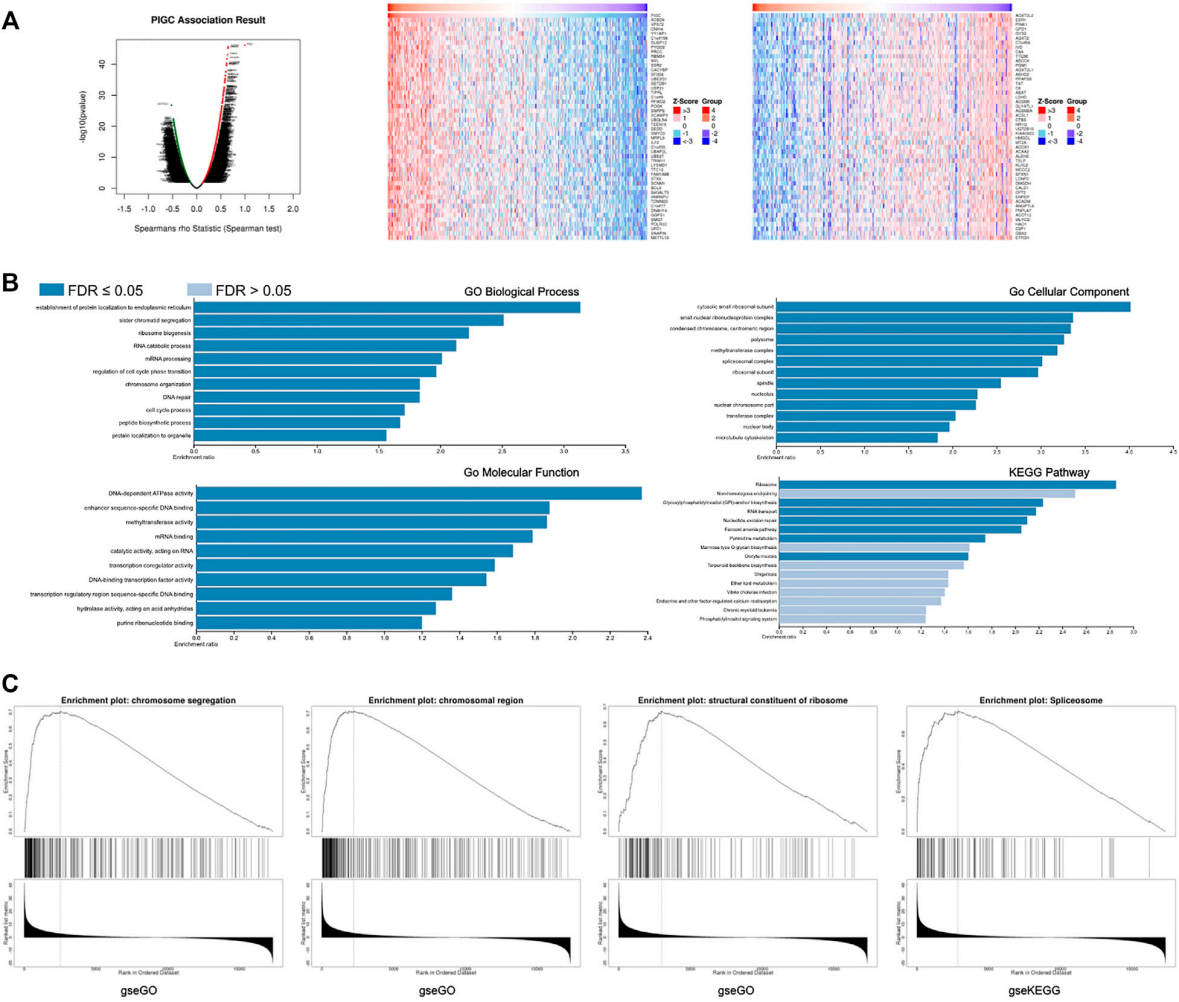
The co-expression of genes with *PIGC* in HCC **(A)** The global PIGC highly correlated genes identified by Pearson test in LIHC cohort. Significant positive correlations highlighted in red, significant negative correlations in green. And the heat maps showing top 50 genes positively and negatively correlated with PIGC in LIHC. Red indicates positively correlated genes and blue indicates negatively correlated genes. **(B)**Co-expressed genes profile with the *PIGC* gene involved in signaling pathways in HCC via ORA tool. **(C)** Co-expressed genes profile with the *PIGC* gene involved in signaling pathways in HCC via GSEA tool.

Next, we investigated genes that were co-expressed with *PIGC* in HCC using LinkedOmics. Figure 6A shows a heat map of the top 50 genes that were positively and negatively correlated with *PIGC* expression. *PIGC* expression showed a strong positive association with the expression of ACBD6 (positive rank#1, r = 0.6622, *p* < 0.001), VPS72 (r = 0.6501, *p* < 0.001), and YY1AP1 (r = 0.6384, *p* < 0.001). Furthermore, *PIGC* expression levels showed a strong negative association with AGXT212 expression levels (r = -0.4911, *p* < 0.001), CPT2 expression levels (r = -0.4818, *p* < 0.001), and HMGCL expression levels (r = -0.4796, *p* < 0.001).

And, GO analysis was performed with *PIGC* using the ORA tool to analyze functionality in biological processes, cellular components and molecular functions. *PIGC* and positively correlated genes were mainly related to the establishment of protein localization to endoplasmic reticulum, cytosolic small ribosomal subunit, and DNA-dependent ATPase activity, respectively. KEGG pathway analysis revealed enrichment in the ribosome(Figure 6B). With regards to GSEA analysis, *PIGC* and positively correlated genes were mainly related to the regulation of chromosome segregation, chromosomal region, structural constituents of the ribosomes and spliceosome(Figure 6C).

### Correlation between *PIGC* and the tumor immune microenvironment

**FIGURE 7:**
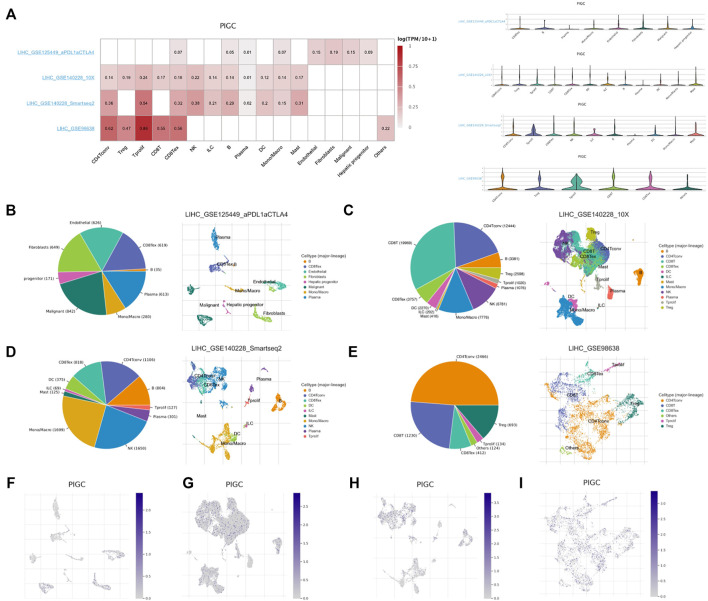
Correlation between *PIGC* and the tumor immune microenvironment using TISCH. **(A)** Average expression of *PIGC* in different cell types, and the distribution of *PIGC* expression in different cell types using violin plot. **(B–E)** The cell types and their distribution in LIHC_GSE125449, LIHC_GSE140228_10X, LIHC_GSE140228_smartseq2 and LIHC_GSE98638 datasets. **(F–I)** Distribution of *PIGC* in different cells in LIHC_GSE125449, LIHC_GSE140228_10X, LIHC_GSE140228_smartseq2 and LIHC_GSE98638 datasets.

We used four datasets (LIHC_GSE125449, LIHC_GSE140228_10X, LIHC_GSE140228_smartseq2 and LIHC_GSE98638) of the TISCH database to evaluate *PIGC* expression in TME-related immune cells. In LIHC_GSE98638 dataset, *PIGC* expression level remains the highest in tprolif cells, CD4Tconv and CD8Tex cells. In LIHC_GSE140228_smartseq2 dataset, *PIGC* expression level remains the highest in tprolif cells, CD4Tconv and NK cells. In LIHC_GSE140228_10X dataset, *PIGC* expression level remains the highest in tprolif cells, NK cells and treg cells. In three datasets, *PIGC* expression level remains the highest in tprolif cells. Violin plot showed the same trend of *PIGC* expression in the HCC cell microenvironment (Figure 7A). *PIGC* expression level in LIHC_GSE125449 is obviously different with other database, maybe because of the immunotherapy effect from LIHC_GSE125449.

In LIHC_GSE125449, malignant cells exhibited the most abundant cell counts (*n* = 842) (Figure 7B). In LIHC_GSE140228_10X, CD8T cells exhibited the most abundant cell counts (*n* = 19969) (Figure 7C). In LIHC_GSE140228_smartseq2, mono/macro cells exhibited the most abundant cell counts (*n* = 1,699) (Figure 7D). In LIHC_GSE98638, CD4Tconv cells exhibited the most abundant cell counts (*n* = 2,466) (Figure 7E). Figures 7F–I represented the distribution of various immune cells related to Figures 7B–E. These results suggest that *PIGC* expression level was quite different in distinct cell types, which might be the source of HCC microenvironment heterogeneity.

### *PIGC* expression was correlated with the level of immune infiltration in HCC

**FIGURE 8:**
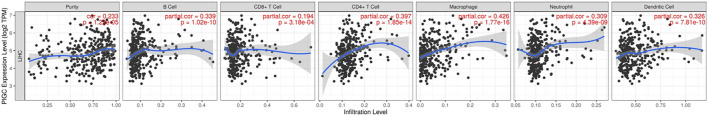
Correlation of *PIGC* expression with immune infiltration level in HCC. *PIGC* expression is significantly positively related to tumor purity and has significant positive correlations with infiltrating levels of B cells, CD8^*+*^ T cells, CD4^*+*^ T cells, macrophages, neutrophils, and dendritic cells in COAD.

Tumor-infiltrating lymphocytes are an independent predictor of sentinel lymph node status and survival in cancer. Therefore, we investigated whether *PIGC* expression was correlated with immune infiltration levels in HCC. The expression levels of *PIGC* were significantly and positively correlated with the infiltrating levels of B cells (r = 0.339, *p* < 0.001), CD8^+^ T cells (*r* = 0.194, *p* < 0.001), CD4^+^ T cells (*r* = 0.397, *p* < 0.001), macrophages (*r* = 0.426, *p* < 0.001), neutrophils (*r* = 0.309, *p* < 0.001) and DCs (*r* = 0.326, *p* < 0.001) in HCC (Figure 8).

Then, we investigated the correlation between *PIGC* expression levels and the status of tumor-infiltrating immune cells based on the gene expression levels of immune marker genes in HCC using the TIMER and GEPIA databases. Tumor purity is an important factor that influences the analysis of immune infiltration in clinical tumor samples by genomic approaches; TIMER and GEPIA feature homologous data from TCGA. We selected cancer types in which *PIGC* expression levels had a significant positive correlation with tumor purity in TIMER and a significant correlation with prognosis in GEPIA.

The immune cells analyzed in HCC tissues included CD8^+^ T cells, CD4^+^ T cells, B cells, (TAMs), monocytes, M1 and M2 macrophages, neutrophils, DCs, and natural killer (NK) cells. Moreover, different subsets of T cells, namely, T helper 1 (Th1), Th2, follicular helper T (Tfh), Th17, regulatory T (Tregs), and exhausted T cells were also analyzed. Since the tumor purity of clinical samples influences the analysis of immune infiltration, the correlation analysis was adjusted for purity. Specifically, *PIGC* expression showed significant correlations with the expression levels of markers of specific immune cells such as T cell markers, CD3D (r = 0.225; *p* < 0.001), CD3E (r = 0.229; *p* < 0.001), and CD2 (r = 0.210; *p* < 0.001); B cell markers, CD19 (r = 0.276; *p* < 0.001), and CD79A (r = 0.222; *p* < 0.001); monocyte markers, CD86 (r = 0.333; *p* < 0.001), and CD115 (r = 0.250; *p* < 0.001); TAM markers, CCL2 (r = 0.214; *p* < 0.001), and CD68 (r = 0.222; *p* < 0.001); M1 macrophage markers, IRF5 (r = 0.480; *p* < 0.001), and COX2 (r = 0.265; *p* < 0.001); neutrophil markers, CD11b (r = 0.288; *p* < 0.001), and CCR7 (r = 0.246; *p* < 0.001); DC markers, HLA-DPB1 (r = 0.230; *p* < 0.001), HLA-DRA (r = 0.262; *p* < 0.001), HLA-DPA1 (r = 0.243; *p* < 0.001), BDCA-1 (r = 0.281; *p* < 0.001), BDCA-4 (r = 0.447; *p* < 0.001), and CD11c (r = 0.326; *p* < 0.001); Th1 markers, STAT1 (r = 0.461; *p* < 0.001), and TNF-α (r = 0.269; *p* < 0.001); Th2 markers, GATA3 (r = 0.238; *p* < 0.001), STAT6 (r = 0.307; *p* < 0.001), and STAT5A (r = 0.329; *p* < 0.001); Tfh marker, BCL6 (r = 0.331; *p* < 0.001); Th17 marker, STAT3 (r = 0.253; *p* < 0.001); Treg markers, CCR8 (r = 0.394; *p* < 0.001), STAT5B (r = 0.415; *p* < 0.0016), and TGFβ (r = 0.393; *p* < 0.001); exhausted T cells markers, PD-1 (r = 0.281; *p* < 0.001), CTLA4 (r = 0.270; *p* < 0.001), and TIM-3 (r = 0.312; *p* < 0.001). These findings strongly suggested that *PIGC* expression correlates with the infiltration of immune cells in HCC (Table 2). Analysis of the GEPIA databases showed that *PIGC* expression in HCC tissues were significantly correlated with the expression of marker genes from tumor infiltrating M1 macrophages, Th2 cells, Tfh cells, and Treg cells (Table 3).

**TABLE 2:** Correlation analysis between PIGC and relate genes and markers of immune cells in TIMER.

Description	Gene marker	HCC			
		NONE		Purity	
		Cor	p	Cor	P
CD8^+^ T cell	CD8A	0.049	0.344	0.190	**
	CD8B	-0.005	0.929	0.128	0.017
T cell (general)	CD3D	0.073	0.162	0.225	***
	CD3E	0.043	0.410	0.229	***
	CD2	0.034	0.512	0.210	***
B cell	CD19	0.170	*	0.276	***
	CD79A	0.053	0.304	0.222	***
Monocyte	CD86	0.146	*	0.333	***
	CD115(CSF1R)	0.074	0.155	0.250	***
TAM	CCL2	0.063	0.227	0.214	***
	CD68	0.098	0.058	0.222	***
	IL10	0.067	0.198	0.200	**
M1 Macrophage	INOS(NOS2)	0.094	0.070	0.100	0.063
	IRF5	0.455	***	0.479	***
	COX2(PTGS2)	0.100	0.054	0.265	***
M2 Macrophage	CD163	-0.023	0.661	0.103	0.055
	VSIG4	0.033	0.521	0.177	**
	MS4A4A	0.015	0.775	0.168	0.002
Neutrophils	CD66b (CEACAM8)	-0.030	0.565	(0.005)	0.927
	CD11b (ITGAM)	0.173	**	0.288	***
	CCR7	0.063	0.229	0.246	***
Natural killer cell	KIR2DL1	-0.092	0.077	(0.115)	0.033
	KIR2DL3	0.145	*	0.201	**
	KIR2DL4	0.080	0.124	0.136	0.011
	KIR3DL1	-0.012	0.810	0.022	0.688
	KIR3DL2	0.067	0.199	0.147	*
	KIR3DL3	-0.008	0.877	(0.012)	0.831
	KIR2DS4	-0.003	0.958	(0.006)	0.906
Dendritic cell	HLA-DPB1	0.072	0.167	0.230	***
	HLA-DQB1	0.015	0.767	0.159	*
	HLA-DRA	0.105	0.042	0.262	***
	HLA-DPA1	0.088	0.090	0.244	***
	BDCA-1(CD1C)	0.147	*	0.281	***
	BDCA-4(NRP1)	0.404	***	0.447	***
	CD11c (ITGAX)	0.162	*	0.326	***
Th1	T-bet (TBX21)	-0.009	0.867	0.119	0.027
	STAT4	0.097	0.063	0.189	**
	STAT1	0.380	***	0.461	***
	IFN-γ (IFNG)	0.095	0.069	0.205	**
	TNF-α (TNF)	0.117	0.025	0.269	***
Th2	GATA3	0.062	0.232	0.238	***
	STAT6	0.318	***	0.307	***
	STAT5A	0.242	***	0.329	***
	IL13	0.004	0.941	0.018	0.739
Tfh	BCL6	0.331	***	0.331	***
	IL21	0.043	0.407	0.098	0.069
Th17	STAT3	0.198	**	0.253	***
	IL17A	0.102	0.049	0.112	0.038
Treg	FOXP3	0.089	0.087	0.146	*
	CCR8	0.266	***	0.394	***
	STAT5B	0.448	***	0.415	***
	TGFβ (TGFB1)	0.242	***	0.393	***
T cell exhaustion	PD-1 (PDCD1)	0.148	*	0.281	***
	CTLA4	0.127	0.015	0.270	***
	LAG3	0.096	0.066	0.174	*
	TIM-3(HAVCR2)	0.121	0.019	0.312	***
	GZMB	0.008	0.875	0.084	0.121

HCC:hepatocellular carcinama; TAM: tumor-associated macrophage; Th: T helper cell; Tfh: Follicular helper T cell; Treg, regulatory T cell; Cor, R value of Spearman’s correlation; None, correlation without adjustment. Purity, correlation adjusted by purity. *p < 0.05, **p < 0.01, ***p < 0.001.

**TABLE 3:** Correlation analysis between PIGC and marker genes of immune cells in GEPIA.

Description	Gene markers	HCC			
		Tumor		Normal	
		R	p	R	P
M1 Macrophage	IRF5	0.45	***	0.68	***
	COX2(PTGS2)	0.13	*	0.49	***
Th2	GATA3	0.088	0.091	0.43	**
	STAT6	0.32	***	0.76	***
	STAT5A	0.26	***	0.7	***
Tfh	BCL6	0.33	***	-0.036	8.00E-01
Th17	STAT3	0.2	***	-0.018	9.00E-01
Treg	CCR8	0.25	***	0.37	**
	STAT5B	0.45	***	0.58	***
	TGFβ (TGFB1)	0.21	***	0.52	***

*p < 0.05, **p < 0.01, ***p < 0.001.

### Drug sensitivity and immunotherapy response analysis

**FIGURE 9:**
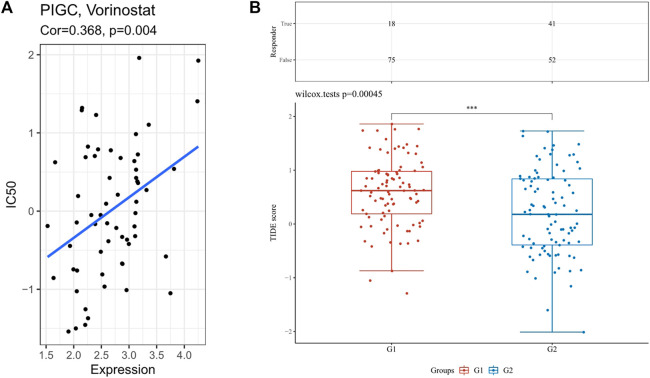
The analysis of drug sensitivity and immunotherapy response via R package **(A)** The correlation between the *PIGC* expression with the IC50. **(B)** TIDE score in different *PIGC* expression levels.

We estimated IC50 values via R package for drug sensitivity evaluations of several chemotherapeutics drugs and compared among different *PIGC* expression. As shown in Figure 9A, the estimated IC50 values of Vorinostat were significantly increased with higher *PIGC* expression, implying that might be less sensitive to the drug (r = 0.368; *p* = 0.004). Futhermore, we assessed the immunotherapy responses of the different *PIGC* levels via TIDE algorithm. The higher *PIGC* expression group (Group 1) has the higher TIDE score (*p* < 0.001) (Figure 9B).

## Discussion

HCC remains one of the major causes of cancer-related deaths worldwide. Hepatocarcinogenesis is a complex multistep process driven by chronic hepatitis that alters the hepatic microenvironment. The number of deaths is proportional to the global incidence, thus highlighting the aggressive tumor biology and the lack of effective therapies. Identifying new targets for the treatment of HCC is very important. The multistep sequence of epigenetic and genetic alterations in the pathogenesis of liver cancer disrupts core cellular processes such as proliferation, cell death, and genome maintenance. In a manner similar to the phenotypic heterogeneity of HCC, the landscape of molecular alterations in HCC is quite extensive. To gain more detailed insights into the potential functions of *PIGC* in HCC and its regulatory network, we performed bioinformatics analysis using public data to guide future research in HCC.

The present systematic clearly demonstrated the prognostic value of *PIGC* in HCC. In our study, we found that the expression levels of *PIGC* are upregulated in brain and CNS, cervical, esophageal, head and neck, and liver cancer, along with myeloma and sarcoma. Furthermore, the highest expression levels of *PIGC* was greatest in liver cancer according to Oncomine, GEPIA and TIMER analyses. Furthermore, the expression levels of *PIGC* mRNA and protein were significantly higher in HCC than in normal tissues. These findings indicated a relationship between *PIGC* and HCC. Additional findings showed that *PIGC* expression was significantly elevated in HCC patients irrespective of sample type, gender, age, race, cancer stage and tumor grade. In addition, high expression levels of *PIGC* were significantly related to OS, RFS, PFS, and DFS. Our study also found that mutations in *PIGC* in HCC patients were associated with patient prognosis. Thus, our results suggest that the upregulation of *PIGC* occurs in many cases of HCC and deserves further clinical validation as a potential diagnostic and prognostic marker.

To investigate alterations in *PIGC* related pathways in HCC, we also analyzed genes that were co-altered along with *PIGC*. Of the positively correlated genes analyzed in the LinkedOmics database, the expression levels of ACBD6 showed the greatest co-alteration with *PIGC* expression. ACBD6 is an acyl-CoA binding protein that is expressed in hematopoietic tissues and appears to be restricted to primitive stem cells present in such tissues (Soupene et al., 2008). Our results provide evidence that ACBD6 proteins play an important role in the myristoylation of proteins in eukaryotic cells (Soupene and Kuypers, 2019). Myristoylation-AKT appears to reverse the effect of CDHR2 and can inhibit the proliferation of HCC cells. (Xia et al., 2019; Wang et al., 2021a). Furthermore, the expression levels of ACBD6 in HCC samples were increased when compared to paired normal tissues according to GEPIA2 analysis. It is possible that *PIGC* can influence the growth of HCC cells via the ACBD6-Myristoylation-AKT pathway. Of the negatively correlated genes determined by the LinkedOmics databse, PINK1 expression showed the highest co-alterations with *PIGC* expression. PINK1 is a serine/threonine kinase that can be imported into the mitochondrial inner membrane via the outer/inner mitochondrial membrane translocase complex, and can be degraded by mitochondrial processing peptidase and mitochondrial inner protease presenilin associated rhomboid like (PARL) (Jin et al., 2010; Lazarou et al., 2012). Recent insights in mitophagy suggest that PINK1 and an E3 ubiquitin ligase (Parkin) play a central role in the quality control of mitochondria (Mancias and Kimmelman, 2016). The role of PINK1-Parkin-mediated mitophagy in the regulation of cell death is the source of much debated and results tend to depend upon the specific context (Zheng et al., 2021).

In addition, we utilized Enrich web tools to identify pathways associated with genes that showed the most correlation with *PIGC* in HCC. From a functional classification viewpoint, the results revealed that the ribosome was associated with the occurrence and progression of HCC. Ribosomes are responsible for the translation of information contained in mRNAs into functional proteins, the ultimate step in the genetic programme. The hyperactivation of ribosome biogenesis, which can be initiated by oncogenes or the loss of tumour suppressor genes has a critical role in cancer initiation and progression (Pelletier et al., 2018).

Besides, the identical result was identifed in the GSEA. The most highly correlated pathway was activation of the splicesome in the tumor; this process represents a key step in gene expression and enables an individual to encode multiple proteins; this process is emerging as a major driver of abnormal phenotypic heterogeneity. It is expected that splicing acts as a potential major source of untapped molecular targets in precision oncology and cancer disparities (Robinson et al., 2019). The spliceosome is a dynamic cellular machine composed of small nuclear ribonucleoproteins (snRNPs) and their associated protein cofactors, and reads information related to the splicing of each pre-mRNA transcript, and is probably the most complicated RNA-protein complex inside eukaryotic cells (Gregory Matera and Wangday, 2014; Paschalis et al., 2018). A number of recent studies have highlighted the fact that mutations and copy-number changes affecting the spliceosomal proteins of key cancer-associated genes are enriched in cancer (Lee and Abdel-Wahab, 2016). Alterations in the splicing of mRNA is emerging as a potentially important driver of cancers; the dysregulation of splicing can give rise to protein isoforms that contribute to tumor establishment, progression, and resistance to therapy (Dvinge et al., 2016; Goodall and Wickramasinghe, 2021).

Cancer development is highly associated to the physiological state of the TME (Roma-Rodrigues et al., 2019). To clarify how *PIGC* might affect TME, we used TISCH single cell database. We observed different immune cell distribution based on HCC sites. In LIHC_GSE140228 dataset and LIHC_GSE98638 dataset, HCC without treatment exhibited relatively high PIGC expression levels in tprolif cells, CD8Tex cells and CD4Tconv compared to expression level in LIHC_GSE125449 dataset. Higher *PIGC* expression was observed in fibroblasts cells in LIHC_GSE125449 dataset, in which HCC patients were on immunotherapy. Therefore, tprolif cells play an outstanding role in TME of HCC, and immunotherapy reshapes tumor microenvironment in patients with HCC.

Another important aspect of this study is that *PIGC* expression was correlated with diverse immune infiltration levels in HCC. Some successful and excellent prognostic models of liver cancer had published, which revealed that the expression of gene such as ARID1A, RBPs is closely related to tumor immune cell infiltration in HCC (Wang et al., 2021b; Feng et al., 2022). Our results demonstrate that there are strong and positive correlations between *PIGC* expression and the infiltration levels of T cells, B cells, and monocytes, and a moderate to strong positive relationship between *PIGC* expression and the infiltration levels of TAM and DCs. This suggests that *PIGC* plays an important role in regulating tumor immunity, and therefore could influence the prognosis of HCC.

Notably, we observed a correlation between the levels of *PIGC* mRNA and the expression of the M1 macrophage marker, IRF5. Furthermore, significant correlations were detected between *PIGC* expression and the levels of several markers of T helper cells (Th2, Tfh, Treg and Th17) in HCC. These correlations could be indicative of a potential mechanism by which *PIGC* regulates the functions of T cells in HCC. The presence of Th2 cells is most typically associated with aggressive tumors. In patients with pancreatic cancer, Th2-type inflammation has been associated with shorter survival, in which tumor-infiltrating Th2 cells correlated with the *in situ* fibroblast, thymic, and stromal production of lymphopoietin. Th2 cell polarization is thought to further drive Th2-type inflammation and lead to dismal outcomes for patients with pancreatic cancer (De Monte et al., 2011). In addition, the present study showed that Th17 cells, a newly defined subset of T helper cells with potent pro-inflammatory properties, are concentrated within HCC tumors and associated with high mortality and reduced survival in HCC patients. Furthermore, the levels of Th17 were positively correlated with the density of microvessels in tumors. The correlations between *PIGC* expression and Th17 indicates that *PIGC* take parts in the inflammatory reaction in HCC and promotes the growth and progression of tumors (Zhang et al., 2009). Our results also indicated that *PIGC* has the potential to activate Treg cells. An increase in *PIGC* expression was positively correlated with the expression of Treg cells; these cells are a subtype of CD4^+^ T cells that suppress anti-self-immune responses (Schmetterer et al., 2012) which can inhibit the development of anti-tumor immunity, thereby hindering the immune surveillance of cancer and preventing effective anti-tumor immune responses in tumor-bearing hosts. By producing cytokines, immunosuppressive Treg cells might promote tumor initiation and development in a chronic inflammatory state; furthermore, high levels of Treg cells are related to a poor OS in HCC (Sun et al., 2017). It is possible that *PIGC* may play an important role in HCC with Tregs by influencing the microenvironment (Togashi et al., 2019). Collectively, our findings suggest that *PIGC* plays an important role in the recruitment and regulation of immune infiltrating cells in HCC, which also play a key role in the poor prognosis of patients with HCC.

Accurate measurement of drug sensitivity and resistance is the cornerstone of cancer biology, pharmacology, and many fundamental studies on cell signaling and cell division. Drug sensitivity and resistance are conventionally quantified by IC50 (Hafner et al., 2016). Our study found that the *PIGC* expression level could affect Vorinostat drug sensitivity. Similarly, HCC patients with high *PIGC* levels had higher TIDE scores, indicating that tumor patients might low respond to immunotherapy. Our study shows that high levels of *PIGC* expression maybe affect the drug sensitivity of HCC patients and affect their benefit from immunotherapy.

Our study has some limitations that need to be considered. Firstly, our investigations into the role of *PIGC* in tumors were based on data that was already reported in the public databases. However, we did not verify these outcomes by testing our own clinical samples. Secondly, we did not conduct *in vitro* or animal experiments to confirm the role of *PIGC* in the growth and progression of HCC and its relationship with the infiltration of immune cells into the tumor microenvironment. Hence, further studies are now needed to verify the role played by *PIGC* in HCC.

## Conclusion

In summary, our results suggest that *PIGC* is a potential independent prognostic biomarker for HCC that can be used to evaluate the levels of immune cell infiltration in tumor tissues. Relatively low levels of *PIGC* in HCC and other cancer tissues may indicate a greater risk of tumor relapse after treatment and careful medical supervision will be necessary for such patients.

## Data Availability

The original contributions presented in the study are included in the article/supplementary material further inquiries can be directed to the corresponding author.
